# Structural and Functional Hallmarks of Sindbis Virus Proteins: From Virion Architecture to Pathogenesis

**DOI:** 10.3390/ijms26178323

**Published:** 2025-08-27

**Authors:** Qibin Geng, Chanakha K. Navaratnarajah, Wei Zhang

**Affiliations:** 1 Institute for Molecular Virology, University of Minnesota-Twin Cities, Minneapolis, MN 55455, USA; 2 Department of Diagnostic and Biological Sciences, School of Dentistry, University of Minnesota-Twin Cities, Minneapolis, MN 55455, USA; 3 Department of Molecular Medicine, Mayo Clinic, Rochester, MN 55905, USA; 4 Mayo Clinic Graduate School of Biomedical Sciences, Mayo Clinic, Rochester, MN 55905, USA; 5 Masonic Cancer Center, University of Minnesota-Twin Cities, Minneapolis, MN 55455, USA; 6 Characterization Facility, College of Sciences and Engineering, University of Minnesota-Twin Cities, Minneapolis, MN 55455, USA

**Keywords:** Sindbis virus, alphavirus, envelope glycoprotein, capsid protein, structural, biology, cryo-electron microscopy, X-ray crystallography, genotypes, pathogenesis

## Abstract

Sindbis virus (SINV), a prototype of the Alphavirus genus (family *Togaviridae*), is a globally distributed arbovirus causing febrile rash and debilitating arthritis in humans. Viral structural proteins—capsid (C), E1, and E2—are fundamental to the virion’s architecture, mediating all stages from assembly to host cell entry and pathogenesis, thus representing critical targets for study. This review consolidates the historical and current understanding of SINV structural biology, tracing progress from early microscopy to recent high-resolution cryo-electron microscopy (cryo-EM) and X-ray crystallography. We detail the virion’s precise *T* = 4 icosahedral architecture, composed of a nucleocapsid core and an outer glycoprotein shell. Key functional roles tied to protein structure are examined: the capsid’s dual capacity as a serine protease and an RNA-packaging scaffold that interacts with the E2 cytoplasmic tail; the E1 glycoprotein’s function as a class II fusion protein driving membrane fusion; and the E2 glycoprotein’s primary role in receptor binding, which dictates cellular tropism and serves as the main antigenic target. Furthermore, we connect these molecular structures to viral evolution and disease, analyzing how genetic variation among SINV genotypes, particularly in the E2 gene, influences host adaptation, immune evasion, and the clinical expression of arthritogenic and neurovirulent disease. In conclusion, the wealth of structural data on SINV offers a powerful paradigm for understanding alphavirus biology. However, critical gaps persist, including the high-resolution visualization of dynamic conformational states during viral entry and the specific molecular determinants of chronic disease. Addressing these challenges through integrative structural and functional studies is paramount. Such knowledge will be indispensable for the rational design of next-generation antiviral therapies and broadly protective vaccines against the ongoing threat posed by SINV and related pathogenic alphaviruses.

## 1. Sindbis Virus: From Discovery to Molecular Pathogenesis

Sindbis virus (SINV), first isolated in 1952 from *Culex univittatus* mosquitoes near Sindbis, Egypt, represents the prototype species of the Alphavirus genus within the family *Togaviridae* [1]. Following its initial discovery, the first recognized human cases emerged in Uganda (1961), South Africa (1963), and Australia (1967), establishing SINV as a globally distributed arbovirus causing febrile rash and debilitating arthritis [1,2]. As the prototype alphavirus, SINV has served as the foundational model for elucidating alphavirus replication strategies, structural protein synthesis, and virion assembly mechanisms [2,3].

**Figure 1:**
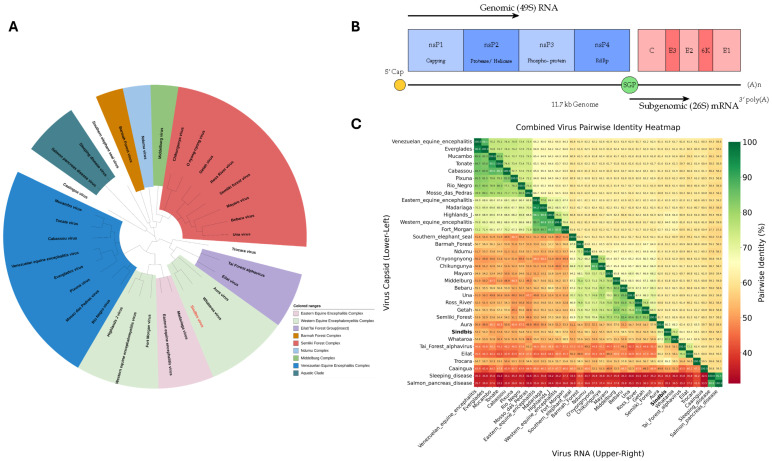
Sindbis virus: phylogenetic context, genomic architecture, and sequence conservation within the alphavirus genus. (**A**) Phylogenetic relationship of Sindbis virus within the *Alphavirus* genus. The unrooted maximum likelihood tree, based on whole-genome alignments, positions Sindbis virus (highlighted in red) among other medically relevant alphaviruses. The tree was generated using the RaxML algorithm with a GTR model from the Bacterial and Viral Bioinformatics Resource Center (BV-BRC) and iTOL v7 tool. (**B**) Schematic of the Sindbis virus (SINV) genome organization. The positive-sense, single-stranded RNA genome of approximately 11.7 kb contains two main open reading frames (ORFs). The 5′-proximal ORF is translated from the genomic RNA (49S) to produce the non-structural polyprotein (nsP1-nsP4). The 3′-proximal ORF is translated from 26S subgenomic RNA (sgRNA) transcribed from the subgenomic promoter (SGP) to produce the structural polyprotein (C, E3, E2, 6K, and E1). (**C**) Pairwise Identity Heatmap. A heatmap displaying a comparison of the percent identity between various alphaviruses. The lower-left triangle compares the amino acid sequences of the capsid proteins, while the upper-right triangle compares the nucleotide sequences of the full-length RNA genomes. The color scale indicates the percentage of identity, from low (red, *∼*30%) to high (green, 100%), highlighting the relative conservation of the capsid protein compared to the overall genomic sequence across the genus.

Phylogenetically, SINV belongs to the Western Equine Encephalitis (WEE) serocom-plex and shares evolutionary relationships with Chikungunya virus and other arthritogenic alphaviruses [1,4,5,6]. As illustrated in Figure 1A, SINV clusters among Old World alphaviruses within the broader phylogenetic landscape of medically relevant species. The virus exhibits considerable genetic diversity, with at least six distinct genotypes (SINV-I to SINV-VI), displaying up to 22.2% amino acid divergence in the E2 glycoprotein [7]. These genotypes show distinct geographical clustering: SINV-I encompasses European and African strains associated with human disease outbreaks, SINV-II and SINV-III include Australian and East Asian isolates with 12–15% E2 divergence from SINV-I, SINV-IV comprises Azerbaijani and Chinese strains, SINV-V represents New Zealand isolates, and the recently proposed SINV-VI includes African-European variants [7,8,9]. The pairwise identity analysis shown in Figure 1C demonstrates the relative conservation of capsid proteins compared to overall genomic sequences across the alphavirus genus. The SINV genome comprises approximately 11.7 kilobases of positive-sense, single-stranded RNA organized into two principal open reading frames, as depicted in Figure 1B [3]. The structural proteins—capsid (C), E3, E2, 6K, and E1—are translated from 26S subgenomic RNA and undergo precisely regulated proteolytic processing initiated by the capsid protein’s serine protease activity [10]. Mature virions exhibit *T* = 4 icosahedral quasisymmetry with 240 copies each of C, E1, and E2 proteins, forming distinct nucleocapsid and glycoprotein shells [10].

Structural understanding has evolved dramatically from early transmission electron microscopy to the high-resolution techniques detailed in Table 1. Landmark achievements include early cryo-electron microscopy reconstructions [9], progressive resolution improvements to 9 Å and 7 Å [10,11,12], crystal structures of the E1-E2 spike complex [13], and the recent 3.5 Å cryo-EM structure, which provides near-atomic-level detail [14]. These structural advances, combined with capsid protein crystallography [15,16,17], have revealed the molecular architecture underlying viral function.

Genotypic variations profoundly impact viral function and pathogenesis. E2 protein differences between strains directly influence mosquito infectivity, with specific motifs at positions 95–96 and 116–119 enhancing *Aedes aegypti* infection efficiency [22]. Mutations in untranslated regions affect host-specific replication and necessitate adaptive changes in structural proteins [23,24]. Specific E2 substitutions can confer heparan sulfate-dependent infection capabilities and alter neuroinvasiveness in animal models [25,26].

Clinically, SINV infection manifests as Pogosta disease, Ockelbo disease, or Karelian fever, characterized by maculopapular rash, arthralgia affecting the wrists, hips, knees, and ankles, and potential progression to chronic arthritis that can last months to years [1]. The structural proteins mediate pathogenic processes through tissue tropism determination, with E2 receptor-binding domains directing cellular specificity and viral replication in periosteum, tendons, and muscle tissues [27,28]. Immune evasion mechanisms include capsid-mediated interleukin-1 receptor-associated kinase 1 (IRAK1) inhibition, E1/E2 glycan shielding, and E2 epitope drift under antibody pressure [29,30,31]. This integrated understanding of SINV structure, diversity, and pathogenesis provides the foundation for rational therapeutic development and represents a paradigm for comprehending alphavirus biology and designing targeted interventions against these important human pathogens.

## 2. Sindbis Virus Architecture and Structural Protein Organization

Sindbis virus virions are spherical, enveloped particles with a diameter of approximately 68 to 70 nm, exhibiting the characteristic *T* = 4 icosahedral quasisymmetry that defines alphavirus’s architecture [4,10]. This sophisticated structural organization dictates that 240 copies each of capsid (C) protein, E1 glycoprotein, and E2 glycoprotein are incorporated into each virion, with E1 and E2 heterodimerizing to form 80 trimeric spikes that protrude from the virion’s surface [10]. The maintenance of this precise symmetry across two structurally distinct protein layers—the outer glycoprotein shell and the inner nucleocapsid core—requires highly orchestrated interactions established primarily through the specific binding between the E2 cytoplasmic domain and a hydrophobic pocket on the capsid protein’s surface [10].

**Figure 2:**
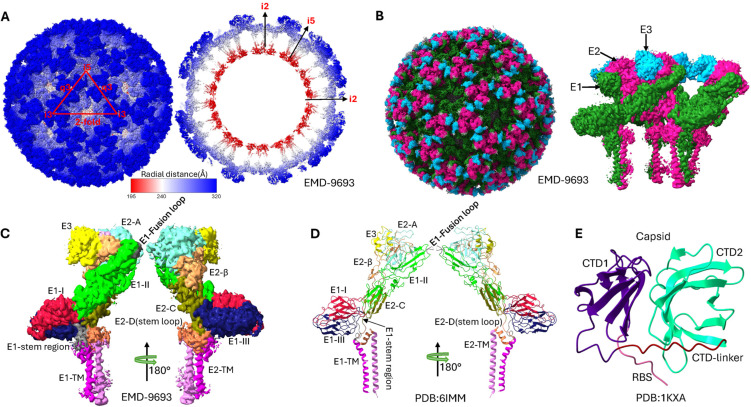
Structural organization and domain architecture of Sindbis virus revealed by cryo-electron microscopy. (**A**) Radial distance coloring of the SINV virion, showing the layered architecture from the nucleocapsid core (inner, red) to the glycoprotein spikes (outer, blue). The color gradient represents increasing distance from the viral center. (**B**) Domain-based coloring of the complete SINV particle, highlighting the distinct glycoprotein shells: E3 (blue), E2 (magenta), and E1 (green). (**C**) Detailed subdomain organization of the E1 and E2 glycoproteins in the cryo-EM density map (EMD-9693), showing the three-dimensional arrangement of functional domains within the spike complex. Domain I (DI), Domain II (DII), Domain III (DIII), and transmembrane domain (TM) of E1 are color-coded, along with domains A, B (missing in this map), C, D, and TM of E2. (**D**) Atomic model representation of the E1/E2 glycoprotein heterodimer (PDB: 6IMM) with subdomain coloring corresponding to the cryo-EM density. The model shows the detailed molecular architecture of the spike proteins including transmembrane regions and their configurations. (**E**) Structural model of the capsid protein core (derived from PDB 1KXA). The C-terminal protease domain (structured) and N-terminal RNA-binding domain (flexible, except the ribosome binding site(RBS)) can be distinguished by their colors.

The SINV virion comprises a multi-layered architecture with two nested icosahedral protein shells separated by a host-derived lipid membrane, as illustrated in Figure 2. The innermost nucleocapsid core consists of genomic RNA tightly packaged by 240 capsid proteins, surrounded by the viral envelope containing embedded E1 and E2 glycoproteins forming the surface spikes [10,32]. The host-derived nature of this lipid envelope significantly influences virion properties, with differences in lipid composition between mammalian and insect cell-derived virions affecting membrane fluidity and potentially modulating glycoprotein spike conformation and infectivity [33,34].

The structural proteins encoded by the 26S subgenomic mRNA undergo precise proteolytic processing to yield functionally distinct components with complementary roles in viral assembly, entry, and pathogenesis, as summarized in Table 2. The capsid protein (264 amino acids, 30 kDa) exhibits a bipartite structure comprising an intrinsically disordered N-terminal RNA-binding domain (residues 1–113) rich in basic amino acids and a well-structured C-terminal domain (residues 114–264) adopting a chymotrypsin-like serine protease fold [10,15]. This dual architecture enables the capsid protein to perform multiple critical functions: activates serine protease activity, which mediates autocatalytic cleavage at the conserved Trp-Ser junction through a classical catalytic triad involving Ser215; specifically recognizes and binds the genomic RNA packaging signal within the nsP1 coding region with nanomolar affinity; and assembles the nucleocapsid core through oligomerization motifs [15,35,36].

The E1 glycoprotein (439 amino acids, 47–50 kDa) functions as the class II viral fusion protein responsible for mediating pH-dependent membrane fusion during endosomal entry [10,18]. Its ectodomain exhibits the characteristic three-domain organization of class II fusion proteins: a central Domain I, an elongated Domain II containing the conserved fusion loop at its distal tip, and an immunoglobulin-like Domain III connecting to the transmembrane anchor [10]. E1 molecules are arranged tangentially to the viral surface, forming an icosahedral scaffold underneath the E2 glycoproteins, and undergo dramatic conformational changes upon low pH exposure, dissociating from E2, inserting the fusion loop into target membranes, and trimerizing into stable rod-like structures that drive membrane fusion through hairpin-like refolding [10,13].

**Figure 3:**
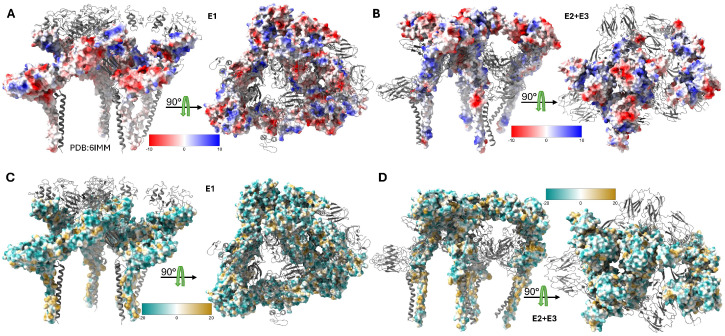
Electrostatic and lipophilic properties of Sindbis virus envelope glycoproteins. Surface representations of the SINV structural proteins derived from the high-resolution cryo-EM structure (PDB: 6IMM) showing distinct physicochemical properties. (**A**) Coulombic electrostatic potential (ESP) map of the E1 glycoprotein, with colors ranging from red (negative potential, −10 kcal/mol*·*e) and white (neutral) to blue (positive potential, +10 kcal/mol*·*e). The ESP calculation used a distance-dependent dielectric (*ε* = 4*d*) with an offset of 1.4 Å from the molecular surface. (**B**) Coulombic electrostatic potential map of the E2 and E3 glycoproteins showing the charge distribution across the receptor-binding and fusion-regulation domains. The same color scale and computational parameters as those in panel A were applied. (**C**) Molecular lipophilicity potential (MLP) map of the E1 glycoprotein, with colors ranging from dark cyan (hydrophilic, −20) and white (neutral) to dark goldenrod (lipophilic, +20). The MLP was calculated using the Fauchere method (*e*^−*d*^) with a 5.0 Å distance cutoff. (**D**) Molecular lipophilicity potential map of the E2 and E3 glycoproteins revealing hydrophobic and hydrophilic regions important for membrane interactions and protein folding.

The E2 glycoprotein (423 amino acids, 50–52 kDa) serves as the primary determinant of cellular tropism and the principal target for neutralizing antibodies [37,38]. As demonstrated in Figure 3, the E2 ectodomain exhibits distinct electrostatic and lipophilic properties across its functional Domains A, B (missing in this map), C, and D, with Domain A containing key receptor-binding determinants, Domain B forming the spike apex and housing neutralizing epitopes, and Domain C mediating extensive E1 interactions [14,20]. E2 recognizes multiple host cell attachment factors including heparan sulfate through positively charged residues and specific entry receptors, such as natural resistance-associated macrophage protein (NRAMP), with the region spanning amino acids 170–220 serving as a critical receptor-binding domain [25,39,40].

**Figure 4:**
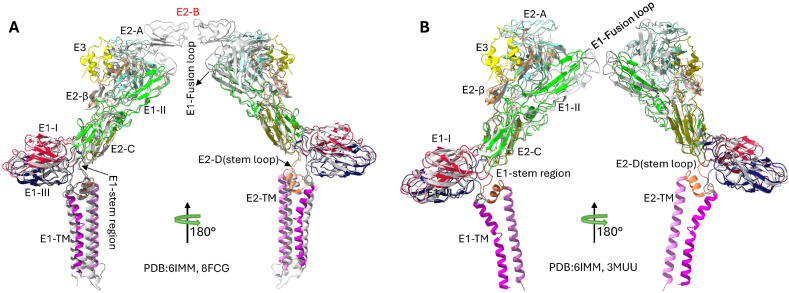
Structural comparisons of Sindbis virus envelope glycoproteins revealing Domain B is missing and pH-induced conformational changes are present. (**A**) Comparative analysis of SINV and CHIKV E1/E2 glycoprotein structures, highlighting E2 Domain B is missing in SINV. The SINV’s structure (PDB: 6IMM) is shown with domain-based coloring consistent with Figure 2, while the CHIKV’s structure (PDB: 8FCG) [41] is displayed in grey. The superposition reveals that SINV lacks the well-ordered E2 Domain B structure present in CHIKV, which normally caps the E1 fusion loop. Domain A, C, and D of E2 are clearly resolved in both structures, demonstrating the conserved overall architecture despite the missing Domain B density in SINV. The RMSD between the structures is 1.178 Å. (**B**) pH-dependent structural comparison of SINV envelope glycoproteins showing conformational changes during the fusion process. The physiological pH structure (PDB: 6IMM, pH 8.0, colored) is compared with the low-pH fusion intermediate structure (PDB: 3MUU, pH 5.6, grey). At low pH levels, significant conformational rearrangements occur, particularly in the E2 domain organization and the *β*-ribbon connector region. The low-pH structure represents an early fusion intermediate where E2 Domain B becomes disordered and detaches from the E1 fusion loop, exposing it for membrane insertion. The RMSD between structures is 1.005 Å, and a 68-degree rotation reflects the conformational changes that occur below the fusion threshold (pH 6.0). These structural transitions are critical for alphavirus membrane fusion and viral entry into host cells.

Structural comparisons reveal important architectural features and the dynamic properties of the SINV envelope proteins, as shown in Figure 4. The absence of well-ordered E2 Domain B density in SINV contrasts with other alphaviruses like Chikungunya virus [41], potentially reflecting the structural flexibility important for receptor binding and fusion regulation [14]. pH-induced conformational changes between physiological (up to a pH of 8.0) and fusion-triggering conditions (a pH of 5.6) demonstrate the dramatic structural rearrangements occurring during viral entry, particularly the disordering of E2 Domain B and the exposure of the E1 fusion loop [13].

E1 and E2 glycoproteins contain conserved N-linked glycosylation sites that critically influence viral function and immune evasion. E1 possesses glycans at Asn139 and Asn245, with differential accessibility leading to complex-type processing at N139 and high-mannose retention at N245, both essential for proper folding, transport, and virulence in vertebrate and invertebrate hosts [30]. E2 glycosylation occurs at Asn196 and Asn318, with these modifications contributing to immune evasion through glycan shielding, while paradoxically enhancing heparan sulfate binding and virulence when eliminated [30,42].

**Table 2:** Overview of Sindbis virus structural proteins and their therapeutic targets.

Protein	Size (Approx. aa/kDa)	Key Functions	Key PTMs	Location in Virion	Antibodies/Inhibitors	Mechanism of Action
Capsid (C)	264 aa/ 30 kDa	Genome packaging, nucleocapsid formation, serine protease activity (autocleavage), interaction with E2 cytoplasmic tail, IRAK1 inhibition	Autoproteolytic cleavage	Nucleocapsid core	**Small molecules [43,44,45,46]:** Berberine Chloride (BBC), Picolinic Acid (PCA) Mandelic Acid (MDA), Ethyl 3-aminobenzoate Dioxane derivatives, Piperazine derivatives **Protease inhibitors [47,48]:** Various serine protease inhibitors	BBC: Perturbs NC assembly/disassembly PCA: Binds CTD hydrophobic pocket, inhibits budding MDA/EAB: High binding affinity to hydrophobic pocket (in silico) Protease inhibitors: Target catalytic triad (H141, D147, S215)
E1	439 aa/ 47–50 kDa	Class II membrane fusion, forms icosahedral scaffold with E2, heterodimerization with E2	N-glycosylation (N139, N245), palmitoylation	Envelope spike (base/scaffold)	**Monoclonal antibodies [49,50,51]:** Sin-33 (neutralizing) Anti-E1 MAbs **Protein inhibitors:** Exogenous E1 DIII domain [52,53] **Small molecules [54,55,56,57]:** Zinc (Zn^2+^), NH_4_Cl/Bafilomycin A1	Sin-33: Induces non-infectious conformation, blocks low-pH changes E1 DIII: Binds fusion intermediate, blocks endogenous DIII fold-back Zn^2+^: Blocks DIII/stem fold-back (H333 interaction) pH modulators: Prevent low-pH conditions that trigger E1 activation
E2	423 aa/ 50–52 kDa	Receptor binding, cell attachment, heterodimerization with E1, interaction with capsid via cytoplasmic tail, major antigen	N-glycosylation (N196, N318), palmitoylation, cleavage from pE2	Envelope spike (surface exposed)	**Monoclonal antibodies [58,59,60,61,62]:** Anti-E2 broadly neutralizing antibodies (bNAbs) R6, R13 (conformational epitope) DC2.M16 and DC2.M357 (Domain B-specific) **Small molecules [63,64]:** Doxycycline, Obatoclax (OLX)	bNAbs: Target conserved epitopes across alphaviruses Domain-specific MAbs: Block receptor binding Doxycycline: Computational binding to E2, impairs conformational changes OLX: Neutralizes endosomal pH
E3	64-65 aa/ 7–8 kDa	Signal sequence for pE2-6K-E1 translocation, chaperone for pE2 folding, pH protection of E1 during transport, processing from pE2	Cleavage from pE2	Mostly released; not a major component of mature SINV	**Limited targets:** Anti-E3 antibodies [65] (rare) Furin inhibitors [66] (indirect)	Furin inhibitors: Block pE2 → E2 + E3 processing, prevent spike maturation
6K	55 aa/6 kDa	Viroporin, membrane permeabilization, glycoprotein processing and transport, budding	None identified	Primarily cellular, very low in virions	**Viroporin inhibitors [67,68]:** Rimantadine analogs, Amiloride derivatives Ion channel blockers	Block viroporin activity, membrane permeabilization Inhibit viral protein transport Reduce budding efficiency
TF	frameshift of 6K/ 8–10 kDa	Virus assembly and release, virulence factor	Palmitoylation	Virion (low amounts)	**Limited information [69]:** Palmitoylation inhibitors Assembly disruptors	Block palmitoylation essential for membrane association Disrupt assembly functions

Data compiled from structural and functional studies [3,10,15,70,71]. Antibody and inhibitor information derived from experimental studies and computational predictions. MAb = Monoclonal antibody; bNAb = Broadly neutralizing antibody; TF = Transframe protein; CTD = C-terminal domain; DIII = Domain III; PTMs = Post-translational modifications; EAB=Ethyl 3-aminobenzoate.

**Figure 5:**
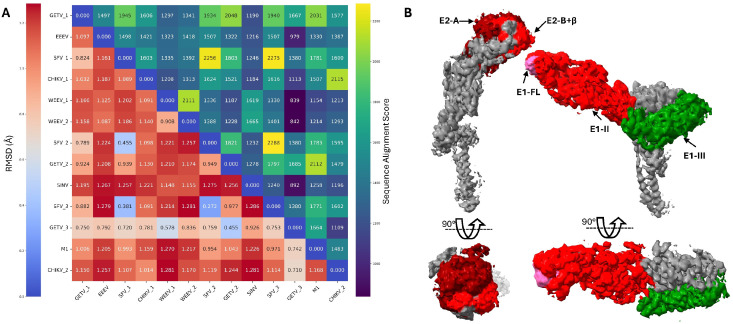
Representative alphavirus structural comparison and antigenic epitope mapping of SINV envelope glycoproteins. (**A**) Combined heatmap displaying structural similarity (RMSD values, lower-left triangle) and sequence alignment scores (upper-right triangle) among representative alphavirus structures. The analysis includes the following: GETV_1 (PDB: 7FD2, Getah virus), EEEV (PDB: 8UFA, Eastern Equine Encephalitis virus), SFV_1 (PDB: 8YVY, Semliki Forest virus), CHIKV_1 (PDB: 8FCG, Chikungunya virus), WEEV_1 (PDB: 9DQX, Western Equine Encephalitis virus), WEEV_2 (PDB: 8DEE, Western Equine Encephalitis virus), SFV_2 (PDB: 8YVZ, Semliki Forest virus), GETV_2 (PDB: 7WC2, Getah virus), SINV (PDB: 6IMM, Sindbis virus), SFV_3 (PDB: 8YW0, Semliki Forest virus), GETV_3 (PDB: 7WCO, Getah virus), M1 (PDB: 7V4T, Alphavirus M1), and CHIKV_2 (PDB: 6NK5, Chikungunya virus). Color scale represents sequence alignment scores from low (purple, <1000) to high (yellow, >2000) for the upper triangle, and low RMSD values in the lower triangle indicate higher structural similarity. (**B**) Antigenic epitope mapping on SINV envelope glycoproteins E1 and E2 showing major antibody binding sites (EMD-9693). E2 Domain A (dark red) and the invisible E2 Domain B (red, not visible due to structural flexibility) represent primary neutralizing epitopes targeted by broadly neutralizing antibodies [58]. E1 Domain II (red) contains critical epitopes including the fusion loop (FL, pink), which are recognized by pan-protective antibodies [72]. E1 Domain III (green) itself can be used as a fusion inhibitor, while also serving as a target for fusion inhibitors and representing a potential therapeutic intervention site [52,53]. The structural organization reflects the accessibility and immunodominance of these regions during natural infection and vaccination.

A comparative structural analysis across multiple alphavirus species reveals both conserved architectural features and sequence divergence, which influence immunogenicity and therapeutic targeting strategies [58,72]. As illustrated in Figure 5A, RMSD analysis and sequence alignment scores demonstrate the structural conservation of envelope glycoproteins across diverse alphaviruses, with SINV serving as the prototype to understand the shared mechanisms of membrane fusion and antibody recognition. The structural similarities support the development of broadly protective interventions, while sequence differences in key epitope regions drive the specificity of neutralizing antibody responses.

The antigenic landscape of SINV envelope glycoproteins reveals distinct epitope clusters that serve as targets for therapeutic intervention (Figure 5B). E2 Domain A contains the primary receptor-binding determinants [73,74] and may represent a major target for neutralizing antibodies [60,75], while the flexible E2 Domain B, though structurally disordered in many high-resolution structures, harbors conserved epitopes recognized by broadly neutralizing antibodies across multiple arthritogenic alphaviruses [58]. The E1 glycoprotein presents complementary targeting opportunities, with Domain II epitopes, particularly surrounding the fusion loop, serving as sites for pan-protective antibody recognition [72]. Notably, E1 Domain III functions not only as an immunogenic region but also as a target for fusion inhibitors, offering a dual therapeutic approach combining passive immunization with direct antiviral intervention [52,53].

The E3 protein (64–65 amino acids, 7–8 kDa) functions as both a signal sequence directing pE2-6K-E1 translocation into the endoplasmic reticulum and a chaperone facilitating proper spike assembly and pH protection during transport [76,77]. Following furin-mediated cleavage from E2 in the trans-Golgi network, E3 is largely released from SINV virions, contrasting with its retention in other alphaviruses and enabling proper spike maturation, which is essential for infectivity [76,77].

The small membrane proteins 6K and TF arise from overlapping genetic sequences through ribosomal frameshifting, with 6K (55 amino acids, 6 kDa) functioning as a viroporin that modulates cellular calcium homeostasis and membrane permeability to facilitate glycoprotein processing and budding [32,78]. In contrast, TF is incorporated into virions at low levels and undergoes the palmitoylation of N-terminal cysteine residues, directing its plasma membrane localization and distinguishing its fate from the largely excluded 6K protein [32,69].

The therapeutic targeting of SINV structural proteins has revealed multiple intervention strategies, as detailed in the expanded Table 2. The capsid protein presents diverse druggable sites, with small molecules like berberine chloride disrupting nucleocapsid assembly and picolinic acid targeting the hydrophobic pocket essential for E2 interaction [43,44,45,46]. The E1 glycoprotein is targeted by the neutralizing monoclonal antibodies like Sin-33, which induces non-infectious conformations, and by the exogenous E1 Domain III protein that acts as a dominant-negative fusion inhibitor [49,50,51,52,53]. Most prominently, the E2 glycoprotein serves as the primary antigenic target, with neutralizing monoclonal antibodies like R6, R13, DC2.M16, DC2.M357, F5, and 3B4C-4 demonstrating therapeutic efficacy through multiple mechanisms. Broadly neutralizing antibodies targeting conserved E2 epitopes, particularly within Domains A and B, represent promising candidates for pan-alphavirus therapeutic development [58,59,60,61,62].

Critical protein–protein interactions orchestrate virion assembly and function. The capsid protein’s hydrophobic pocket accommodates both intra-capsid N-terminal arm interactions during nucleocapsid assembly and E2 cytoplasmic tail binding through a conserved Tyr-X-Leu motif during budding, with residues Tyr180 and Trp247 mediating these specific interactions [3,15]. The E1-E2 heterodimer formation in the endoplasmic reticulum is essential for proper folding and transport, with E2 acting as a pH-sensitive regulator that prevents premature E1 activation until appropriate endosomal conditions trigger the initiation of the fusion cascade [10,29].

Beyond their structural roles, SINV proteins exhibit sophisticated immune modulation capabilities. The capsid protein inhibits IRAK1 signaling pathways upon cytoplasmic delivery, creating a permissive environment for infection establishment [29]. E2 serves as the primary antigenic target with neutralizing epitopes concentrated in the receptor-binding domain, driving evolutionary pressure for epitope drift, which facilitates immune evasion while maintaining its receptor recognition capability [7,31].

The quasisymmetric architecture creates subtle conformational heterogeneity among chemically identical protein subunits, particularly affecting E2 molecules at different icosahedral positions, which may influence receptor-binding avidity and spike cooperativity during membrane fusion [14]. This sophisticated structural organization, combining precise symmetry with functional flexibility, exemplifies the evolutionary optimization of alphavirus architecture for effective host cell recognition, entry, and immune evasion across diverse biological environments.

## 3. Future Perspectives: Integrating Structural Knowledge for Therapeutic Advancement

The structural proteins of Sindbis virus—capsid, E1, E2, E3, and 6K/TF—represent a remarkable example of molecular evolution, in which 240 copies each of C, E1, and E2 are meticulously arranged with *T* = 4 icosahedral quasisymmetry to create a sophisticated macromolecular machine [10]. Decades of research employing progressively advanced biochemical, genetic, and structural biology techniques have illuminated the intricate roles these proteins play throughout the viral life cycle, from genome packaging and virion assembly to host cell interaction, entry, and immune modulation. The capsid protein serves dual functions as both a serine protease and RNA-binding scaffold while facilitating budding through interaction with the E2 cytoplasmic tail and providing early immune evasion through IRAK1 inhibition [29]. The E1 glycoprotein operates as a tightly regulated class II fusion protein whose activity is modulated by pH and E2 interaction, while E2 serves as the primary determinant of host cell recognition and a major antigenic target [10]. The auxiliary proteins E3 and 6K/TF contribute essential functions in glycoprotein processing, transport, spike maturation, and membrane permeabilization [32].

The structural variations observed across different SINV genotypes and strains, particularly within the E2 glycoprotein, fundamentally underpin the virus’s global distribution, host adaptation strategies, and diverse pathogenic manifestations ranging from asymptomatic infection to debilitating chronic arthralgia [11]. These structural features, including receptor-binding domains and glycosylation patterns, dictate tissue tropism and disease progression while positioning these proteins at the forefront of immune system interactions through mechanisms such as glycan shielding and epitope drift.

Despite remarkable progress, particularly the achievement of near-atomic-level resolution structures of intact SINV virions, significant knowledge gaps persist that limit our comprehensive understanding of alphavirus biology. The absence of high-resolution structural information for several critical components remains a fundamental challenge. While the 3.5 Å cryo-EM structure of the intact virion provides unprecedented detail [14], atomic-resolution structures of full-length E2 glycoprotein, including its transmembrane and cytoplasmic domains in native spike conformation, remain elusive. The dynamic conformational landscape of the E1/E2 spike complex during receptor binding, pH-triggered activation, and membrane fusion represents another critical gap, as current static structures provide only snapshots of what is inherently a dynamic process. The structural organization of small membrane proteins 6K and TF, their oligomerization mechanisms, and precise interactions with viral and host proteins are largely unknown at the atomic level, complicated by their small size and membrane association. Furthermore, while the capsid protein arrangement is well characterized, the precise organization of genomic RNA within the nucleocapsid core and specific RNA–protein interactions beyond the primary packaging signal require further definition at a higher resolution.

The molecular mechanisms underlying SINV pathogenesis present another frontier requiring intensive investigation. The precise molecular basis by which structural proteins contribute to chronic arthralgia remains incompletely understood [4], necessitating the identification of specific E2 epitopes or E1/E2 conformations that interact with joint tissue cells and trigger persistent inflammation. The comprehensive mapping of interactions between SINV structural proteins and host factors in both vertebrate and invertebrate systems continues to develop, with recent discoveries such as the role of sorting nexin 5 (SNX5) in alphavirus replication, highlighting the importance of host-directed research [79]. The unexpected detection of the non-structural protein nsP2 within purified SINV virions from multiple host cell types opens entirely new research avenues regarding potential early infection functions [79]. Additionally, the structural determinants governing efficient infection and transmission by different mosquito vector species across all SINV genotypes require further elucidation to predict and control disease outbreaks effectively.

The therapeutic landscape for alphavirus diseases remains notably deficient, with no clinically approved antiviral therapies or vaccines specifically targeting Sindbis virus or most arthritogenic alphaviruses [80]. However, the wealth of structural information now available presents unprecedented opportunities for rational therapeutic development. Entry inhibitors targeting E2 glycoprotein receptor binding or E1-mediated fusion mechanisms represent promising antiviral strategies, with the recently identified hydrophobic pocket in E2 serving as a potential novel target [14]. Assembly and budding inhibitors that disrupt critical interactions between capsid proteins and E2 cytoplasmic tails, or interfere with capsid oligomerization and RNA packaging, offer additional therapeutic avenues [11].

Vaccine development strategies leveraging structural protein knowledge encompass multiple promising approaches. Subunit vaccines utilizing recombinant E1 and E2 proteins or specific domains thereof capitalize on E2’s role as the primary target for neutralizing antibodies [81]. Virus-like particles formed by the expression of SINV structural proteins provide non-infectious platforms displaying native glycoprotein spikes as potent immunogens. SINV replicons are increasingly explored as vaccine vectors for expressing antigens from diverse pathogens, including SARS-CoV-2 and Dengue virus [82], while chimeric SINV-based vaccines incorporating insect-specific alphavirus backbones like Eilat virus offer enhanced safety profiles [83]. The identification of conserved epitopes across multiple arthritogenic alphaviruses, particularly within Domain B of E2, represents a critical goal for developing broadly protective antibodies and pan-alphavirus vaccines [58].

Future research directions will likely emphasize higher-resolution dynamics through advanced structural biology techniques such as time-resolved cryo-electron microscopy and single-molecule FRET to capture conformational transitions during cell entry. In situ structural biology approaches, including cryo-electron tomography and correlative light and electron microscopy, will enable the visualization of virus–protein interactions and assembly processes directly within infected cells. Systems virology methodologies integrating structural data with proteomics, transcriptomics, and functional genomics will contribute to our comprehensive understanding of SINV–host interactions mediated by structural proteins. Structure-guided antiviral and vaccine design will increasingly leverage growing structural databases to rationally engineer novel inhibitors and improved immunogens capable of eliciting broad, durable protective immunity.

The continued development of SINV-based therapeutic platforms, including gene therapy vectors, oncolytic virotherapy applications [84], and vaccine delivery systems, will benefit substantially from a deeper understanding of structural protein functions. Comparative structural pathogenesis studies examining high-resolution structures of different SINV genotypes and strains with varying pathogenic profiles will identify critical structural correlates of virulence and tissue tropism, informing both basic virology and clinical applications.

The foundational research on SINV structural proteins established over decades of investigation continues to provide essential insights into alphavirus biology while serving as a paradigm for understanding related pathogens. As we advance toward more sophisticated therapeutic interventions, the integration of structural knowledge with emerging technologies and interdisciplinary approaches will be paramount for addressing the ongoing global health threat posed by Sindbis virus and related pathogenic alphaviruses. The remarkable architectural precision of the SINV virion, combined with our evolving understanding of its functional complexities, positions this system as both a model for fundamental virology research and a platform for innovative therapeutic development as part of continuing efforts to combat arboviral diseases.

## Figures and Tables

**Table 1:** Selected PDB and EMDB structures of Sindbis virus and related alphavirus structural proteins/virions.

ID	Protein(s)/Complex	Organism	Res. (Å)	Method	Key Relevance/Reference
**Sindbis Virus Capsid Protein (C)**					
1KXA	C (106–264)	SINV	3.10	X-ray	C-term domain, protease fold [15]
1KXF	C (1–264, C-term ordered)	SINV	2.38	X-ray	C-term chymotrypsin-like [15]
1SVP	C (mutant, C-term)	SINV	2.00	X-ray	N-term arm binding pocket [16]
1WYK	C (114–264)	SINV	2.00	X-ray	Pocket conformation study [17]
2SNW	C (C-term)	SINV	2.70	X-ray	Type3 crystal form [15]
**Sindbis Virus Glycoproteins (E1, E2, E3) and Virions (Cryo-EM)**					
1LD4	Virion (E1, E2, C fitted)	SINV	11.4	Cryo-EM	Early *T* = 4 model, E1/E2 shape [10]
EMD-1121	Virion (TE12 E2-N318Q)	SINV	9.0	Cryo-EM	E2 topology, spike differences [11]
1Z8Y	E1/E2 model (fitted)	SINV	9.0	Model (EM)	E2 topology from EMD-1121 [11]
3J0F	Virion (C, E1, E2 fitted)	SINV	7.0	Cryo-EM	Model of cdE2-CP interactions [12]
3MUU	E1-E2 spike (low pH)	SINV	3.29	X-ray	Fusion intermediate structure [13]
3MUW	E1-E2 shell (model)	SINV	9.0	Model (EM)	Pseudo-atomic E1/E2 shell [13]
6IMM	Virion (E1, E2, E3)	SINV	3.5	Cryo-EM	High-res virion, E2 pocket [14]
EMD-9693	Virion map for 6IMM	SINV	3.5	Cryo-EM	High-res virion map [14]
**Semliki Forest Virus (SFV) Glycoproteins (Homologs for E1/E2)**					
1I9W	E1 ectodomain	SFV	3.0	X-ray	Class II fusion protein fold [18]
2ALA	E1 ectodomain (monomer)	SFV	3.0	X-ray	Monomeric E1 structure [19]
		SFV,			
8X0L	Virion with VLDLR	*Homo sapiens*	3.5	Cryo-EM	SFV-receptor complex [20]
1RER	E1 ectodomain (trimer)	SFV	3.3	X-ray	Post-fusion trimer [21]

## References

[1] European Centre for Disease Prevention and Control (ECDC) (2025). Facts About Sindbis Fever. https://www.ecdc.europa.eu/en/sindbis-fever/facts.

[2] Strauss J.H., Strauss E.G. (1994). The alphaviruses: Gene expression, replication, and evolution. Microbiol. Rev..

[3] Jose J., Snyder J.E., Kuhn R.J. (2009). A structural and functional perspective of alphavirus replication and assembly. Future Microbiol..

[4] Harding S., Sewgobind S., Johnson N. (2023). JMM Profile: Sindbis virus, a cause of febrile illness and arthralgia. J. Med. Microbiol..

[5] Lavergne A., de Thoisy B., Lacoste V., Pascalis H., Pouliquen J.F., Mercier V., Tolou H., Dussart P., Morvan J., Talarmin A. (2006). Mayaro virus: Complete nucleotide sequence and phylogenetic relationships with other alphaviruses. Virus Res..

[6] Weaver S.C., Kang W., Shirako Y., Rumenapf T., Strauss E.G., Strauss J.H. (1997). Recombinational history and molecular evolution of western equine encephalomyelitis complex alphaviruses. J. Virol..

[7] Lundström J.O., Pfeffer M. (2010). Phylogeographic structure and evolutionary history of Sindbis virus. Vector Borne Zoonotic Dis..

[8] Ling J., Smura T., Lundström J.O., Pettersson J.H.O., Sironen T., Vapalahti O., Lundkvist A., Hesson J.C. (2019). Introduction and Dispersal of Sindbis Virus from Central Africa to Europe. J. Virol..

[9] Michie A., Ernst T., Pyke A.T., Nicholson J., Mackenzie J.S., Smith D.W., Imrie A. (2023). Genomic Analysis of Sindbis Virus Reveals Uncharacterized Diversity within the Australasian Region, and Support for Revised SINV Taxonomy. Viruses.

[10] Zhang W., Mukhopadhyay S., Pletnev S.V., Baker T.S., Kuhn R.J., Rossmann M.G. (2002). Placement of the Structural Proteins in Sindbis Virus. J. Virol..

[11] Mukhopadhyay S., Zhang W., Gabler S., Chipman P.R., Strauss E.G., Strauss J.H., Baker T.S., Kuhn R.J., Rossmann M.G. (2006). Mapping the Structure and Function of the E1 and E2 Glycoproteins in Alphaviruses. Structure.

[12] Tang J., Jose J., Chipman P., Zhang W., Kuhn R.J., Baker T.S. (2011). Molecular Links between the E2 Envelope Glycoprotein and Nucleocapsid Core in Sindbis Virus. J. Mol. Biol..

[13] Li L., Jose J., Xiang Y., Kuhn R.J., Rossmann M.G. (2010). Structural changes in alphavirus E1 and E2 glycoproteins during membrane fusion. Nature.

[14] Chen L., Wang M., Zhu D., Sun Z., Ma J., Wang J., Kong L., Wang S., Liu Z., Wei L. (2018). Implication for alphavirus host-cell entry and assembly indicated by a 3.5Å resolution cryo-EM structure. Nat. Commun..

[15] Choi H.K., Lee S., Zhang Y.P., McKinney B.R., Wengler G., Rossmann M.G., Kuhn R.J. (1996). Structural analysis of Sindbis virus capsid mutants involving assembly and catalysis. J. Mol. Biol..

[16] Lee S., Owen K.E., Choi H.K., Lee H., Lu G., Wengler G., Brown D.T., Rossmann M.G., Kuhn R.J. (1996). Identification of a protein binding site on the surface of the alphavirus nucleocapsid and its implication in virus assembly. Structure.

[17] Lee S., Kuhn R.J., Rossmann M.G. (1998). Probing the potential glycoprotein binding site of sindbis virus capsid protein with dioxane and model building. Proteins.

[18] Lescar J., Roussel A., Wien M.W., Navaza J., Fuller S.D., Wengler G., Rey F.A. (2001). The Fusion glycoprotein shell of Semliki Forest virus: An icosahedral assembly primed for fusogenic activation at endosomal pH. Cell.

[19] Roussel A., Lescar J., Vaney M.C., Wengler G., Rey F.A. (2006). Structure and Interactions at the Viral Surface of the Envelope Protein E1 of Semliki Forest Virus. Structure.

[20] Li Y., Zhao Z., Liu S., Wang H., Sun J., Chai Y., Zhou J., Wang Y., Shi Y., Song H. (2023). Structural basis of Semliki Forest virus entry using the very-low-density lipoprotein receptor. hLife.

[21] Gibbons D.L., Vaney M.C., Roussel A., Vigouroux A., Reilly B., Lepault J., Kielian M., Rey F.A. (2004). Conformational change and protein-protein interactions of the fusion protein of Semliki Forest virus. Nature.

[22] Pierro D.J., Powers E.L., Olson K.E. (2008). Genetic Determinants of Sindbis Virus Mosquito Infection Are Associated with a Highly Conserved Alphavirus and Flavivirus Envelope Sequence. J. Virol..

[23] Fayzulin R., Frolov I. (2004). Changes of the Secondary Structure of the 5′ End of the Sindbis Virus Genome Inhibit Virus Growth in Mosquito Cells and Lead to Accumulation of Adaptive Mutations. J. Virol..

[24] Garneau N.L., Sokoloski K.J., Opyrchal M., Neff C.P., Wilusz C.J., Wilusz J. (2008). The 3′ Untranslated Region of Sindbis Virus Represses Deadenylation of Viral Transcripts in Mosquito and Mammalian Cells. J. Virol..

[25] Zhu W., Fu S., He Y., Li J., Liang G. (2010). Amino acid substitutions in the E2 glycoprotein of Sindbis-like virus XJ-160 confer the ability to undergo heparan sulfate-dependent infection of mouse embryonic fibroblasts. Virol. J..

[26] Dubuisson J., Lustig S., Ruggli N., Akov Y., Rice C.M. (1997). Genetic determinants of Sindbis virus neuroinvasiveness. J. Virol..

[27] Heise M.T., Simpson D.A., Johnston R.E. (2000). Sindbis-Group Alphavirus Replication in Periosteum and Endosteum of Long Bones in Adult Mice. J. Virol..

[28] Assunção-Miranda I., Cruz-Oliveira C., Da Poian A.T. (2013). Molecular Mechanisms Involved in the Pathogenesis of Alphavirus-Induced Arthritis. Biomed Res. Int..

[29] Jose J., Przybyla L., Edwards T.J., Perera R., Burgner J.W., Kuhn R.J. (2012). Interactions of the cytoplasmic domain of Sindbis virus E2 with nucleocapsid cores promote alphavirus budding. J. Virol..

[30] Knight R.L., Schultz K.L.W., Kent R.J., Venkatesan M., Griffin D.E. (2009). Role of N-Linked Glycosylation for Sindbis Virus Infection and Replication in Vertebrate and Invertebrate Systems. J. Virol..

[31] Myles K.M., Pierro D.J., Olson K.E. (2003). Deletions in the putative cell receptor-binding domain of Sindbis virus strain MRE16 E2 glycoprotein reduce midgut infectivity in Aedes aegypti. J. Virol..

[32] Sanz M.A., Madan V., Nieva J.L., Carrasco L., Fischer W.B. (2005). The Alphavirus 6K Protein. Viral Membrane Proteins: Structure, Function, and Drug Design.

[33] He L., Piper A., Meilleur F., Myles D.A.A., Hernandez R., Brown D.T., Heller W.T. (2010). The Structure of Sindbis Virus Produced from Vertebrate and Invertebrate Hosts as Determined by Small-Angle Neutron Scattering. J. Virol..

[34] Dunbar C.A., Rayaprolu V., Wang J.C.Y., Brown C.J., Leishman E., Jones-Burrage S., Trinidad J.C., Bradshaw H.B., Clemmer D.E., Mukhopadhyay S. (2019). Dissecting the Components of Sindbis Virus from Arthropod and Vertebrate Hosts: Implications for Infectivity Differences. ACS Infect. Dis..

[35] Sokoloski K.J., Nease L.M., May N.A., Gebhart N.N., Jones C.E., Morrison T.E., Hardy R.W. (2017). Identification of Interactions between Sindbis Virus Capsid Protein and Cytoplasmic vRNA as Novel Virulence Determinants. PLoS Pathog..

[36] Owen K.E., Kuhn R.J. (1997). Alphavirus budding is dependent on the interaction between the nucleocapsid and hydrophobic amino acids on the cytoplasmic domain of the E2 envelope glycoprotein. Virology.

[37] Navaratnarajah C.K., Kuhn R.J. (2007). Functional characterization of the Sindbis virus E2 glycoprotein by transposon linker-insertion mutagenesis. Virology.

[38] Snyder A.J., Sokoloski K.J., Mukhopadhyay S. (2012). Mutating Conserved Cysteines in the Alphavirus E2 Glycoprotein Causes Virus-Specific Assembly Defects. J. Virol..

[39] Rose P.P., Hanna S.L., Spiridigliozzi A., Wannissorn N., Beiting D.P., Ross S.R., Hardy R.W., Bambina S.A., Heise M.T., Cherry S. (2011). Natural Resistance-associated Macrophage Protein (NRAMP) is a cellular receptor for Sindbis virus in both insect and mammalian hosts. Cell Host Microbe.

[40] Byrnes A.P., Griffin D.E. (1998). Binding of Sindbis Virus to Cell Surface Heparan Sulfate. J. Virol..

[41] Chmielewski D., Su G.C., Kaelber J.T., Pintilie G.D., Chen M., Jin J., Auguste A.J., Chiu W. (2024). Cryogenic electron microscopy and tomography reveal imperfect icosahedral symmetry in alphaviruses. Proc. Natl. Acad. Sci. USA Nexus.

[42] Tong Y., Lavillette D., Li Q., Zhong J. (2018). Role of Hepatitis C Virus Envelope Glycoprotein E1 in Virus Entry and Assembly. Front. Immunol..

[43] Aggarwal M., Kaur R., Saha A., Mudgal R., Yadav R., Dash P.K., Parida M., Kumar P., Tomar S. (2017). Evaluation of antiviral activity of piperazine against Chikungunya virus targeting hydrophobic pocket of alphavirus capsid protein. Antivir. Res..

[44] Sharma R., Kesari P., Kumar P., Tomar S. (2018). Structure-function insights into chikungunya virus capsid protein: Small molecules targeting capsid hydrophobic pocket. Virology.

[45] Sharma R., Fatma B., Saha A., Bajpai S., Sistla S., Dash P.K., Parida M., Kumar P., Tomar S. (2016). Inhibition of chikungunya virus by picolinate that targets viral capsid protein. Virology.

[46] Wan J.J., Brown R.S., Kielian M. (2020). Berberine Chloride is an Alphavirus Inhibitor That Targets Nucleocapsid Assembly. mBio.

[47] Fatma B., Kumar R., Singh V.A., Nehul S., Sharma R., Kesari P., Kuhn R.J., Tomar S. (2020). Alphavirus capsid protease inhibitors as potential antiviral agents for Chikungunya infection. Antivir. Res..

[48] Aggarwal M., Dhindwal S., Kumar P., Kuhn R.J., Tomar S. (2014). trans-Protease Activity and Structural Insights into the Active Form of the Alphavirus Capsid Protease. J. Virol..

[49] Kim A.S., Kafai N.M., Winkler E.S., Gilliland T.C., Cottle E.L., Earnest J.T., Jethva P.N., Kaplonek P., Shah A.P., Fong R.H. (2021). Pan-protective anti-alphavirus human antibodies target a conserved E1 protein epitope. Cell.

[50] Calvert A.E., Bennett S.L., Hunt A.R., Fong R.H., Doranz B.J., Roehrig J.T., Blair C.D. (2022). Exposing cryptic epitopes on the Venezuelan equine encephalitis virus E1 glycoprotein prior to treatment with alphavirus cross-reactive monoclonal antibody allows blockage of replication early in infection. Virology.

[51] Hernandez R., Paredes A., Brown D.T. (2008). Sindbis Virus Conformational Changes Induced by a Neutralizing Anti-E1 Monoclonal Antibody. J. Virol..

[52] Roman-Sosa G., Kielian M. (2011). The Interaction of Alphavirus E1 Protein with Exogenous Domain III Defines Stages in Virus- Membrane Fusion. J. Virol..

[53] Sánchez-San Martín C., Sosa H., Kielian M. (2008). A Stable Prefusion Intermediate of the Alphavirus Fusion Protein Reveals Critical Features of Class II Membrane Fusion. Cell Host Microbe.

[54] Schuchman R.M., Vancini R., Piper A., Breuer D., Ribeiro M., Ferreira D., Magliocca J., Emmerich V., Hernandez R., Brown D.T. (2018). Role of the vacuolar ATPase in the Alphavirus replication cycle. Heliyon.

[55] Kononchik J.P., Hernandez R., Brown D.T. (2011). An alternative pathway for alphavirus entry. Virol. J..

[56] Hunt S.R., Hernandez R., Brown D.T. (2011). Role of the Vacuolar-ATPase in Sindbis Virus Infection. J. Virol..

[57] Liu C.Y., Kielian M. (2012). Identification of a Specific Region in the E1 Fusion Protein Involved in Zinc Inhibition of Semliki Forest Virus Fusion. J. Virol..

[58] Fox J.M., Long F., Edeling M.A., Lin H., van Duijl-Richter M.K., Fong R.H., Kahle K.M., Smit J.M., Jin J., Simmons G. (2015). Broadly neutralizing alphavirus antibodies bind an epitope on E2 and inhibit entry and egress. Cell.

[59] Malonis R.J., Earnest J.T., Kim A.S., Angeliadis M., Holtsberg F.W., Aman M.J., Jangra R.K., Chandran K., Daily J.P., Diamond M.S. (2021). Near-germline human monoclonal antibodies neutralize and protect against multiple arthritogenic alphaviruses. Proc. Natl. Acad. Sci. USA.

[60] Earnest J.T., Holmes A.C., Basore K., Mack M., Fremont D.H., Diamond M.S. (2021). The mechanistic basis of protection by non-neutralizing anti-alphavirus antibodies. Cell Rep..

[61] Porta J., Jose J., Roehrig J.T., Blair C.D., Kuhn R.J., Rossmann M.G. (2014). Locking and Blocking the Viral Landscape of an Alphavirus with Neutralizing Antibodies. J. Virol..

[62] Pence D.F., Davis N.L., Johnston R.E. (1990). Antigenic and genetic characterization of Sindbis virus monoclonal antibody escape mutants which define a pathogenesis domain on glycoprotein E2. Virology.

[63] Ching K.C., Ng L.F.P., Chai C.L.L. (2017). A compendium of small molecule direct-acting and host-targeting inhibitors as therapies against alphaviruses. J. Antimicrob. Chemother..

[64] Varghese F.S., Rausalu K., Hakanen M., Saul S., KÃijmmerer B.M., Susi P., Merits A., Ahola T. (2017). Obatoclax Inhibits Alphavirus Membrane Fusion by Neutralizing the Acidic Environment of Endocytic Compartments. Antimicrob. Agents Chemother..

[65] Parker M.D., Buckley M.J., Melanson V.R., Glass P.J., Norwood D., Hart M.K. (2010). Antibody to the E3 Glycoprotein Protects Mice against Lethal Venezuelan Equine Encephalitis Virus Infection. J. Virol..

[66] Ozden S., Lucas-Hourani M., Ceccaldi P.E., Basak A., Valentine M., Benjannet S., Hamelin J., Jacob Y., Mamchaoui K., Mouly V. (2008). Inhibition of Chikungunya Virus Infection in Cultured Human Muscle Cells by Furin Inhibitors: Impairment of the Maturation of the E2 Surface Glycoprotein. J. Biol. Chem..

[67] Elmasri Z., Negi V., Kuhn R.J., Jose J. (2022). Requirement of a functional ion channel for Sindbis virus glycoprotein transport, CPV-II formation, and efficient virus budding. PLoS Pathog..

[68] Santos I.A., Pereira A.K.d.S., Guevara-Vega M., de Paiva R.E.F., Sabino-Silva R., Bergamini F.R.G., Corbi P.P., Jardim A.C.G. (2022). Repurposing potential of rimantadine hydrochloride and development of a promising platinum(II)-rimantadine metallodrug for the treatment of Chikungunya virus infection. Acta Trop..

[69] Ramsey J., Renzi E.C., Arnold R.J., Trinidad J.C., Mukhopadhyay S. (2017). Palmitoylation of Sindbis Virus TF Protein Regulates Its Plasma Membrane Localization and Subsequent Incorporation into Virions. J. Virol..

[70] UniProt Consortium (2025). Structural polyprotein—Sindbis virus (SINV). https://www.uniprot.org/uniprotkb/P03316/entry.

[71] Kim K.H., Strauss E.G., Strauss J.H. (2000). Adaptive Mutations in Sindbis Virus E2 and Ross River Virus E1 That Allow Efficient Budding of Chimeric Viruses. J. Virol..

[72] Williamson L.E., Reeder K.M., Bailey K., Tran M.H., Roy V., Fouch M.E., Kose N., Trivette A., Nargi R.S., Winkler E.S. (2021). Therapeutic alphavirus cross-reactive E1 human antibodies inhibit viral egress. Cell.

[73] Tsetsarkin K.A., McGee C.E., Volk S.M., Vanlandingham D.L., Weaver S.C., Higgs S. (2009). Epistatic Roles of E2 Glycoprotein Mutations in Adaption of Chikungunya Virus to *Aedes Albopictus* and *Ae. Aegypti* Mosquitoes. PLoS ONE.

[74] Weger-Lucarelli J., Aliota M.T., Wlodarchak N., Kamlangdee A., Swanson R., Osorio J.E. (2016). Dissecting the Role of E2 Protein Domains in Alphavirus Pathogenicity. J. Virol..

[75] Fong R.H., Banik S.S.R., Mattia K., Barnes T., Tucker D., Liss N., Lu K., Selvarajah S., Srinivasan S., Mabila M. (2014). Exposure of Epitope Residues on the Outer Face of the Chikungunya Virus Envelope Trimer Determines Antibody Neutralizing Efficacy. J. Virol..

[76] Paredes A.M., Heidner H., Thuman-Commike P., Venkataram Prasad B.V., Johnston R.E., Chiu W. (1998). Structural Localization of the E3 Glycoprotein in Attenuated Sindbis Virus Mutants. J. Virol..

[77] Snyder A.J., Mukhopadhyay S. (2012). The alphavirus E3 glycoprotein functions in a clade-specific manner. J. Virol..

[78] Scott C., Griffin S. (2015). Viroporins: Structure, function and potential as antiviral targets. J. Gen. Virol..

[79] Schuchman R., Kilianski A., Piper A., Vancini R., Ribeiro J.M.C., Sprague T.R., Nasar F., Boyd G., Hernandez R., Glaros T. (2018). Comparative Characterization of the Sindbis Virus Proteome from Mammalian and Invertebrate Hosts Identifies nsP2 as a Component of the Virion and Sorting Nexin 5 as a Significant Host Factor for Alphavirus Replication. J. Virol..

[80] Karki D., LaPointe A.T., Isom C., Thomas M., Sokoloski K.J. (2024). Mechanistic insights into Sindbis virus infection: Noncapped genomic RNAs enhance the translation of capped genomic RNAs to promote viral infectivity. Nucleic Acids Res..

[81] Kam Y.W., Lum F.M., Teo T.H., Lee W.W.L., Simarmata D., Harjanto S., Chua C.L., Chan Y.F., Wee J.K., Chow A. (2012). Early neutralizing IgG response to Chikungunya virus in infected patients targets a dominant linear epitope on the E2 glycoprotein. EMBO Mol. Med..

[82] Scaglione A., Opp S., Hurtado A., Lin Z., Pampeno C., Noval M.G., Thannickal S.A., Stapleford K.A., Meruelo D. (2021). Combination of a Sindbis-SARS-CoV-2 Spike Vaccine and Îs’OX40 Antibody Elicits Protective Immunity Against SARS-CoV-2 Induced Disease and Potentiates Long-Term SARS-CoV-2-Specific Humoral and T-Cell Immunity. Front. Immunol..

[83] Hall R.A., Nguyen W., Khromykh A.A., Suhrbier A. (2025). Insect-specific virus platforms for arbovirus vaccine development. Front. Immunol..

[84] Pampeno C., Opp S., Hurtado A., Meruelo D. (2024). Sindbis Virus Vaccine Platform: A Promising Oncolytic Virus-Mediated Approach for Ovarian Cancer Treatment. Int. J. Mol. Sci..

